# Refined carbohydrates and the overfat pandemic: implications for brain health and public health policy

**DOI:** 10.3389/fpubh.2025.1585680

**Published:** 2025-10-28

**Authors:** Philip Maffetone, Paul B. Laursen

**Affiliations:** ^1^Independent Researcher, Ormond Beach, FL, United States; ^2^Auckland University of Technology, Auckland, New Zealand

**Keywords:** refined carbohydrates, added sugar, ultra-processed foods, overfat, insulin resistance, neuroinflammation, cognition, mood

## Abstract

Refined carbohydrate exposure—principally added sugars and rapidly digestible starches—is a modifiable driver of the overfat pandemic and carries downstream risks for brain health. This narrative review synthesizes epidemiological, clinical, and mechanistic evidence linking refined carbohydrates to excess adiposity and metabolic dysfunction, and in turn to cognitive, affective, and addiction-related outcomes. Converging data show that high-glycemic, ultra-processed foods promote positive energy balance via glycemic volatility, impaired satiety signaling, and reinforcement of dopaminergic reward pathways; chronic exposure contributes to insulin resistance, ectopic fat, systemic inflammation, and cerebrovascular burden. These states are associated with reduced executive function, attentional control, mood dysregulation, and heightened compulsive intake. Experimental studies demonstrate short-term effects on craving, reward responsivity, and glycemic variability, while longitudinal cohorts relate higher refined carbohydrate intake and markers of adiposity to poorer cognitive trajectories and greater depression risk. Although other dietary components may influence brain health, this review focuses on refined carbohydrates as a primary, tractable lever. Public health options include front-of-pack warnings for added sugars, targeted taxation and marketing restrictions (especially to children), procurement standards, reformulation targets, school and healthcare environment changes, and screening for overfat as a clinical vital sign. Priorities for research include causal trials that manipulate refined carbohydrate exposure with brain outcomes, and evaluation of policy packages at population scale. Reducing refined carbohydrate exposure offers a plausible, scalable strategy to curb overfat and protect brain health.

## Introduction

Social determinants of individual and population health are critical concepts in public health and often discussed as outcomes influenced by various social factors. Conversely, this paper addresses brain health as a primary factor influencing individual and social health. Brain health encompasses the complete physical, biochemical, and mental–emotional performance of the brain throughout aging, leading to more meaningful, impactful, purposeful, and productive lives that positively influence others and society (1–3). It is the preservation of overall structure and function independent of underlying pathophysiological processes (2, 4). The World Health Organization (WHO) defines brain health as the state of function across cognitive, sensory, social–emotional, behavioral and motor domains, allowing a person to realize their full potential throughout life irrespective of the presence or absence of disorders (Optimizing Brain Health across the Life Course. World Health Organization; Geneva, Switzerland: 2022). As such, the terms *brain health* and *brain function* are used interchangeably here. Also referred to as neurocognitive performance and incorporating mental health, brain health encompasses behavior, decision-making, planning, self-awareness, interactions, and cooperation with others. These factors impact overall health (5), and are dependent on learning, language, and communication which further enhances the brain. Creativity is also vital, facilitating the generation of novel concepts through the improved organization of established principles, furthering successful human achievements (6).

Individual and socioeconomic stress can adversely affect brain health (7). For example, financial fallout from escalating healthcare costs, lost workdays, reduced productivity, and increased disability intensifies economic hardship, compounding risks to cognition, behavior, mental disorders, and impaired social functioning across individuals, families, and populations (8, 9). This can contribute to societal challenges such as aggression and conflict (10).

Early subtle brain impairment can lead to preclinical dysfunction and poor performance predictive of later disease representing an unaddressed social and public health issue requiring immediate attention (11, 12). The WHO projects that by 2040 neurodegenerative diseases will become the second-leading cause of death worldwide (13). Optimizing brain health to ensure individual and societal wellbeing cannot be overstated (3, 4).

Although the brain is often viewed as either healthy or clinically impaired, early unattended reductions in function can occur in otherwise healthy individuals beginning in early life (14, 15). Other studies indicate that a significant proportion of adults meet the criteria for mental health disorders having their onset in childhood (16, 17). Research further highlights widespread executive dysfunction during everyday activities in non-clinical young adults with no psychiatric or neurological history (18, 19). Mild subclinical depression or anxiety often predicts the future clinical occurrence of these disorders (20). Moreover, reduced brain health is evident in asymptomatic subjects aged 50–80, with white matter loss being a significant risk factor for cognitive impairment and dementia (12), even in neurologically symptom-free diabetics (21). In addition, the early development of reduced brain health can accelerate biological aging, a major risk factor leading to increased morbidity and mortality (22).

### A proactive response

Wide-ranging individual and social challenges necessitate healthy brain function to support processes like planning, creativity, communication, self-restraint, reasoning, and empathy, and without them brain dysfunction can contribute to individual and social adversities (23–26). A shift from predominantly reactive brain health care to a balanced emphasis of proactive approaches can more effectively address these serious problems. Proactive care entails early primary prevention to reduce or eliminate risk factors, disease, and premature death, while also maintaining or improving brain function throughout life. Reactive care, on the other hand, focuses on screening for and treating existing disease (3, 27).

As declining brain health often begins subtly and early in life with long preclinical periods spanning decades of accumulating damage before clinical symptoms emerge (28), proactive care represents a critical opportunity to implement a conservative, cost-effective approach to help prevent or delay declines in brain function (2, 27). It can also positively influence quality of life, healthcare, and related economic costs (19, 23, 27, 29–31). While early diagnosis and intervention of disease is still critical, integrating both proactive and reactive strategies is essential for easing these burdens (32, 33) helping to ensure a healthier future for all individuals and societies. It could also positively influence the global burden of non-communicable diseases (NCD), health disparities, environmental and planetary health, social injustices, community breakdown, poverty, and other critical issues that reflect the enduring challenges described across disciplines such as anthropology, economics, public policy, and sociology (34, 35, 295).

Healthy lifestyles can significantly enhance brain function throughout aging, helping achieve individual and social purpose (36). Here we emphasise the important influence of diet on the full spectrum of brain health.

### Diet and brain health

Global food production and dietary habits have changed significantly, especially over the past half-century. It is well known that healthy food contributes to reduced infant mortality, improved life expectancy, disease prevention, and influences brain health beginning in utero and throughout adulthood. However, the food supply has become more unhealthy due to processed items that, beginning early in life, can significantly promote poor brain health, NCD, including heart disease, stroke, some cancers, Type 2 diabetes, and Alzheimer’s disease, and raise healthcare costs (37–41). Specifically, unhealthy foods can contribute to excess body fat (42), termed *overfat*, estimated to affect ~80% of the world’s population (43–45) and also reduced brain health (46–50). Excess body fat is associated with several risk factors that can impact the brain, including early-onset impaired glucose metabolism due to insulin resistance in both the brain and body that can contribute to neurodegeneration and cognitive decline (51–53). Studies indicate that brain glucose hypometabolism is present in individuals at genetic risk for Alzheimer’s disease long before symptoms arise, particularly among those with a maternal family history of the disease (51, 54–56). Depression, even mild forms, is an independent predictor of increased mortality in patients with chronic disease (57).

Other conditions associated with excess body fat that can impair brain health include cardiometabolic dysregulation (58–61). Related abnormalities include chronic inflammation producing proinflammatory cytokines (62, 63) along with mitochondrial dysfunction and increased oxidative stress, further impairing glucose regulation (64). In addition, studies demonstrate impairment of leptin signaling that can induce Alzheimer’s-like pathologies such as *β*-amyloid accumulation and hyperphosphorylation of tau protein (65). Also increasingly evident over the past half-century, reductions in both gray and white matter volumes have been observed in individuals with excess body fat (66–71). This can contribute to early brain atrophy posing significant risks for the onset and progression of neurodegenerative diseases, including Parkinson’s, Alzheimer’s, and multiple sclerosis (68, 72). A primary component of unhealthy diets and common cause of excess body fat is refined carbohydrates.

### Refined carbohydrates

As unhealthy diets are responsible for more deaths globally than any other risks, including tobacco, drugs, alcohol, and unsafe sex combined (41, 73), refined carbohydrates are one of the most significant contributors to an unhealthy diet. The consumption of refined carbohydrates is a common denominator promoting many individual clinical factors that impair neurocognitive function throughout the lifespan in adults and children worldwide (74–85). As discussed below, we use “refined carbohydrates” to include added sugars and refined starches as primary components of unhealthy, fast or junk foods. Virtually all areas of the world have experienced an explosion of refined carbohydrate consumption and associated excess body fat and reduced brain health (43–45, 72, 86).

As food links environmental sustainability, the production and manufacture of unhealthy foods can also affect planetary health through climate change, biodiversity loss, freshwater use, nitrogen and phosphorus cycles, land-system changes, and chemical pollution, contributing to global unhealthy food-related harm to ecosystems and public health (41, 81, 82, 87, 88, 295).

**Figure 1:**
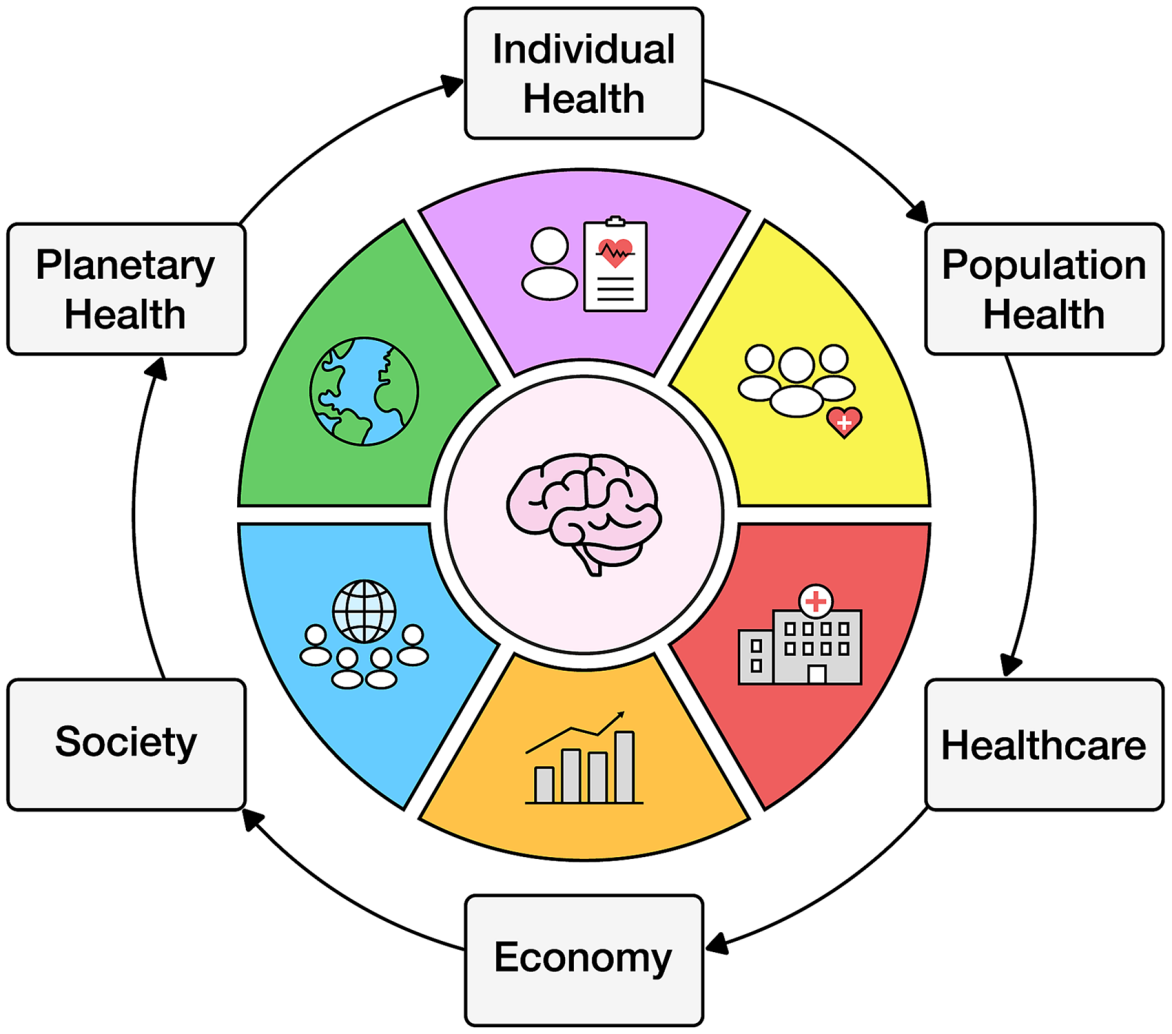
Cycle of individual health behavior affecting population, society, and planetary health.

Individual behavior affects the population which influences healthcare, the economy, society, and the environment (89) (see Figure 1).

### Defining refined carbohydrates

**Figure 2:**
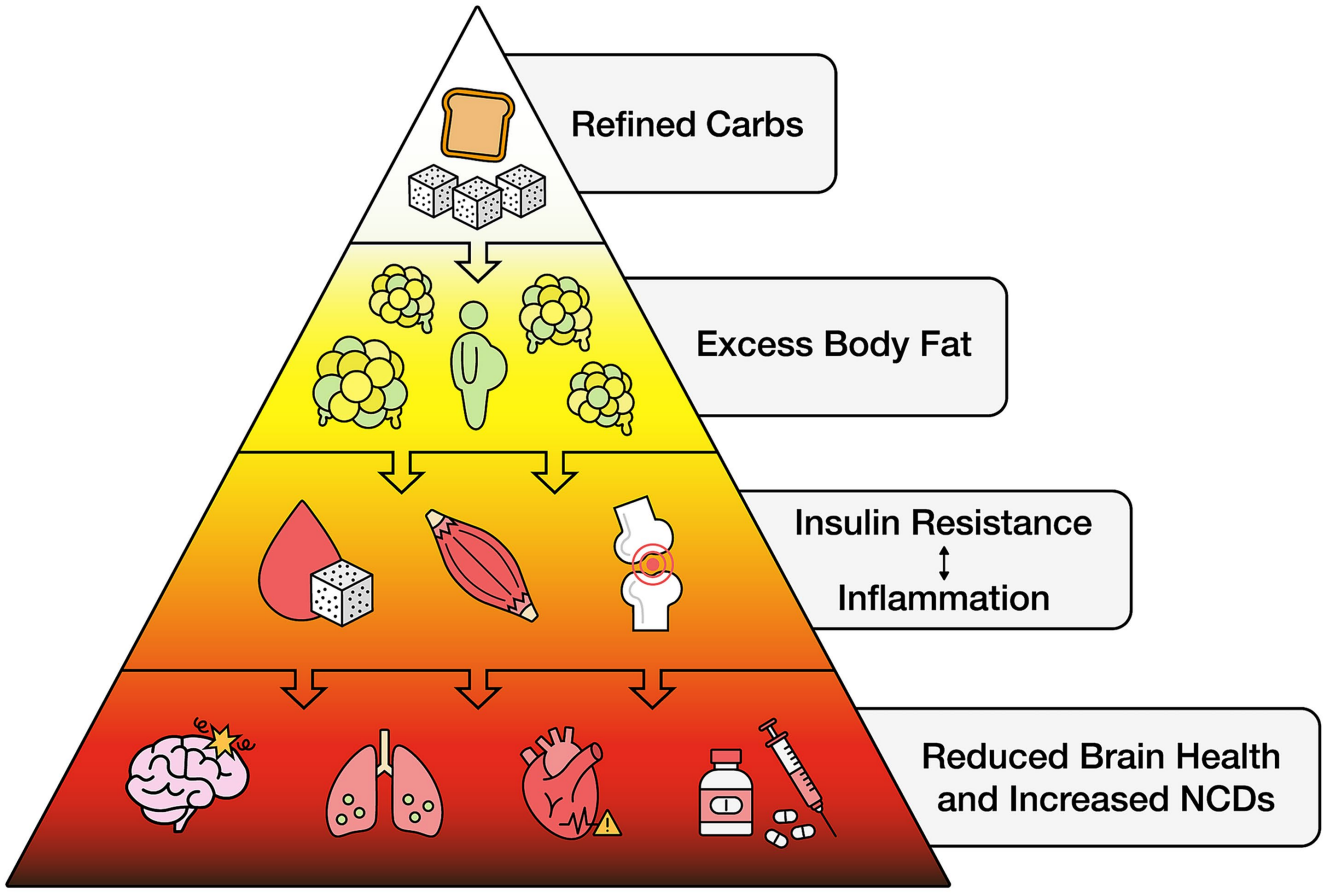
Refined carbohydrates and reduced brain health.

Refined carbohydrates include sugars added to food and drink during processing or manufacturing, or by consumers before consuming them. Included is white and brown cane and beet sugar, molasses and honey, corn, malt, and other syrups, fruit nectars and concentrates, and other foods containing moderate or high levels of glucose and fructose known to potentially reduce health (90). Included are artificial sweeteners, which can induce glucose intolerance by altering gut microbiota (91). The gut-brain axis, a bidirectional communication network between the gastrointestinal tract and brain that incorporates endocrine and immune functions can also be adversely affected by refined carbohydrate consumption (Figure 2).

Refined carbohydrates also include most grain flours common to many foods including wheat, rye, corn, and other ingredients whose starches can quickly convert to glucose and fructose during digestion. Before processing, unrefined whole grains are defined as whole, intact, ground, or cracked, whose starchy endosperm, germ, and bran are present in the same relative portions as they exist in nature (92). However, processing removes the outer bran and inner germ with substantial loss in fiber, B vitamins, iron, magnesium, vitamin E, and other nutrients, making these whole grains no longer whole grains: refined-grain products are nutritionally inferior to their whole-grain counterparts and do not confer the same cardiometabolic and other potential health benefits (93, 94). For example, consuming whole grains can result in less storage of body fat in those consuming approximately ≥3 servings compared with those consuming <1 serving/d, even after accounting for other lifestyle and dietary factors. Moreover, adding refined grains to whole grain products or meals can offset these potential benefits (95).

The advertising and labeling of flour-based food products and ingredients employs various terms and definitions depending on government and regulatory agencies, and often contain high percentages of refined grains despite being labeled as “whole grain,” which can be unclear or confusing to consumers (96, 97). Products containing refined carbohydrates are found in most breads, cereal, pasta, snack foods, soft drinks, condiments, pre-packaged frozen foods, soups and sauces, and other packaged and take-out foods, including many restaurant meals (98).

Separately, the *NOVA* food classification system describes refined carbohydrates as *processed* and *ultra-processed*, and categorized under group 3 and group 4, respectively (99). Group 3 foods undergo significant processing, often including added sugar, while group 4 foods experience chemical modifications that break down whole foods into components with little resemblance to their natural state (100). These products are nutrient-poor, highly degraded, artificially engineered, and often marketed as “healthy” despite contributing to over 30 health conditions (81, 82, 101–103). Also referred to as junk foods, they are high in sugar, found in sweetened beverages, snacks, and even some “plant-based” products designed to mimic the appearance and flavor of animal products (103, 104). Commonly made from highly processed soy, wheat, oat, and other carbohydrates, they are often misrepresented as healthy alternatives but qualify as unhealthy due to their low nutritional value and high levels of added sugars and/or other refined carbohydrates (105). Similar packaged food products marketed for infants and toddlers can also be high in sugar and other refined carbohydrates, displacing natural, nutrient-rich options and contributing to early dietary imbalances (106). All these unhealthy foods and food ingredients are referred to here as refined carbohydrates.

From a health standpoint, restricting the consumption of refined carbohydrates can be a highly effective primary dietary intervention. It can rapidly reduce excess body fat and other related health impairments such as abnormal blood sugar, blood pressure, triglycerides, and reduce the need for related medications (102, 107–109). As refined carbohydrate products for adults and children has significantly increased globally, they are displacing natural and traditional nutrient-dense foods, resulting in lower intakes of micro- and macronutrient intakes; unhealthy foods now the most common cuisine worldwide (110, 111, 296).

Refined carbohydrates exert their detrimental effects through rapid glycemic responses, promoting insulin resistance, visceral fat accumulation, and neurochemical imbalances affecting dopamine and reward systems, and appetite regulation. It is also important to note that natural carbohydrates such as those found in honey, fruits and vegetables, and unrefined starches, while healthy for many, may be unhealthy for individuals with excess body fat, often accompanied by insulin resistance. In addition, while the Healthy Eating Index (HEI) and Diet Quality Index (DQI) are commonly used to assess diet quality in populations, they generally only recommend limiting refined carbohydrate consumption (112, 113).

### Socioeconomic status, brain health, body fat, and refined carbohydrates

To reduce the burden of excess body fat and decreased brain health, it is important to consider social determinants of health—the conditions in which people are born, raised, live, and their age, including socioeconomic status (SES) (114). Broadly defined, SES includes education, income, occupation, perceptions of social status, and access to opportunities and resources, which can influence health and food choices through behavioral and psychological factors (115) with some research showing SES partially mediates observed racial and ethnic disparities (116).

While brain and body health outcomes occur through many complex pathways and physiological mechanisms, a large body of evidence suggests likely causal roles are strongly linked with income and education (117). However, the high prevalence of combined excess body fat, reduced brain health, and increased consumption of refined carbohydrates in virtually all regions of the world make the analysis of SES more difficult. Most importantly, improving brain health and reducing excess body fat can be accomplished with lifestyle modification throughout every individual’s lifespan (118), especially by reducing refined carbohydrate consumption as discussed in this paper.

Consumption of refined carbohydrates is highly prevalent among the full range of socioeconomic groups, including studies demonstrating a more recent sharp increase of unhealthy foods in low- and middle-income countries (119–121). While not exclusively evaluating refined carbohydrates, a systematic assessment of dietary patterns across 187 nations between 1990 and 2010 showed that diet quality varied by age, sex, national income, and region, and in most areas of the world unhealthy food consumption outpaced healthy food with SES and diet quality only weakly correlated (122). In addition, as globalization of unhealthy food progresses, advertising and marketing of these foods significantly increases more populations to abandon their cultural identities concerning traditional healthy food (119, 123). This includes regions of low SES such as sub-Saharan Africa where those exposed to media more likely consume unhealthy foods (124).

Food insecurity is the limited or uncertain ability to acquire adequate food and is experienced globally and associated with reduced brain health, increased body fat, and lower diet quality (125–127). This exposes more low-income populations in developing countries to the same low-cost, refined carbohydrate nutritionally poor foods that make low-income people in the U. S. particularly vulnerable to excess body fat, impaired brain function, and its associated downstream NCD (128, 129). U. S. food assistance programs for low-income families can also contribute to the food insecurity-obesity paradox (130). Overall, as the availability of unhealthy food increases globally, those with low- and moderate-income may consume more of these foods and influence brain and body health (125). However, food insecurity, low SES, and excess body fat are associated in both the general population and across many population subgroups with some studies showing this pattern may be independent of education and income (131, 132).

Other studies show there may be little variation across SES measured as income and wealth, as adults all along this spectrum consume unhealthy food with the middle class eating slightly more than the poor and the wealthy (133), with changes in socioeconomic status during the lifetime also not affecting consumption. This includes the influence of other factors such as age, gender, food accessibility, body fat, physical activity, nutrition knowledge, with overall consumption of refined carbohydrate remaining high (119, 133). In a diverse, low-income population, Hidaka et al. (134) showed high educational attainment (college graduate or higher) increased unhealthy food intake among women but not men. But in general, women tend to eat differently in part because they believe healthy eating is more important but have more difficulty cooking healthy meals, while men prefer the taste of unhealthy food and have reduced self-control.

Globally, overall consumer demand for refined carbohydrate foods greatly depends on price and consumer perception of cost, time for meal preparation, and food preference, with price and food quantity inversely related (135, 136). However, unhealthy food is only perceived as inexpensive with studies showing that healthy, home-prepared meals are often more cost-effective and result in lower energy and sugar intakes, while frequent eating out is associated with significantly higher per capita food expenditures overall (137–139).

While those with economic restraints in developing regions are more inclined to purchase inexpensive unhealthy foods, consumers in developed areas respond similarly. A systemic review and meta-analysis that included primary studies implementing taxes on sugar-sweetened beverages (SSB) in 45 countries with a range of socioeconomic conditions, including the U. S., the U. K., Mexico, Chili, and other nations, it was demonstrated that taxing SSB was associated with higher prices and sustained reductions in sales without significant substitution of untaxed beverages except water, with little differences between socioeconomic status including income (140). Other studies demonstrate that low-income consumers have the highest consumption of SSB (141) that contributes to the obesity epidemics of most developing countries (123, 142–145). Global monitoring of SSB sales is important as decreased use also reduces the consumption of unhealthy meals (133).

While those with lower incomes may spend a smaller portion of it on food, and therefore often choose less expensive items, these behaviors may not apply when purchasing tobacco. As smoking is declining in most developed countries, the inequality of smoking prevalence persists in lower socioeconomic groups (146).

Regardless of SES, a multifaceted approach to reduce refined carbohydrate while improving healthy food intake should address cost, accessibility, and cultural factors, along with education, food assistance programs, and especially focused policy changes described below to help address this global problem.

### The sugar-tobacco connection

Sugar and other refined carbohydrates as a food addiction behavior and tobacco use are both prevalent causes of preventable chronic disease morbidity and mortality worldwide, and a significant healthcare burden (147). There is emerging evidence that these two disorders can develop concurrently or sequentially within individuals, following similar but not identical neurological, physiological, and behavioral abnormalities (148). A greater understanding of how these two disorders are related or overlap is important in addressing their socioeconomic and public health responses, despite research on sugar’s role in food addiction being a relatively new field of study compared to that of tobacco. Both food and tobacco addiction are maladaptive behaviors in which individuals experience compulsive engagement and loss of control despite usually knowing the harmful consequences (149), with tobacco use elevated in those with food addictions. Both nicotine and sugar can alter the brain’s reward pathways through dopamine release, engaging different neural pathways, engendering addictive-like responses in susceptible individuals (150, 151). While sugars are natural tobacco components, they are also frequently added to tobacco during the manufacturing process, contributing to the addictive potential and adverse health effects of tobacco (152, 153). Added sugar also serves as agreeable (to smokers) taste and olfactory sensations, especially in young smokers, and can generate acetaldehyde, which can also be addictive.

Another health problem associated with tobacco use is its relationship to increased body fat: while smokers generally have lower body weight and or body mass index (BMI) they tend to possess more abdominal fat, particularly harmful visceral fat, than non-smokers (154–159). This suggested causal effect of smoking on increased body fat is independent of socioeconomic status and alcohol consumption. Despite nicotine’s effects on appetite reduction and altered metabolism, a population-based, cross-sectional study of 40,036 participants showed no evidence to support the belief that smoking protects against overweight and obesity (158). Smoking may increase body fat through its effect on increased cortisol and reduced sex hormone levels (155).

### Refined carbohydrates and addictive behaviors

On their own, refined carbohydrate foods have been associated with addictive behaviors that negatively impact mental health, contributing to eating disorders, mood disorders, and anxiety disorders (160). This growing individual, social, and public health issue also connects refined carbohydrate consumption, overfat, and reduced brain health (161). While animal studies demonstrate that sugar consumption can lead to behaviors such as bingeing, craving, and withdrawal, which trigger the release of endogenous opioids, these effects bear substantial parallels to the mechanisms of drug addiction as confirmed by human studies (162, 163) (DiNicolantonio et al., 2018). Shared characteristics between drug and food abuse include overconsumption, preoccupation, intense craving, and continued use despite awareness of adverse consequences.

However, not all experts agree with the term “food addiction,” suggesting instead that these issues align more closely with a behavioral condition termed “eating addiction.” Proponents of this view argue that, except for substances like caffeine and alcohol, no evidence suggests that food or its ingredients cause substance-based addictions (164, 165).

### The overfat pandemic

As noted, excess body fat may be a potential early marker of reduced brain health. While determination of body fat content is traditionally accomplished through overweight and obese classifications and measures of BMI, these do not directly assess body fat. There are notable racial and ethnic disparities in excess body fat prevalence, with higher rates observed among Black, Asian, Indigenous, and other non-White populations (43, 44, 166, 167), and despite its widespread use, BMI often misclassifies body composition in these disparities, failing to identify over 50% of individuals with excess body fat and associated disease risk factors (168, 169). The term *overfat* was defined as the presence of excess body fat that negatively impacts physical, biochemical, and mental–emotional health (170). It is estimated that approximately 80% of the world’s population may be overfat (43, 44). Globally, 20–40% of adults classified as having normal weight and BMI may still be overfat (171, 172), a condition often referred to as *normal weight obesity* which has also been linked to cardiometabolic disease and other health risks (173).

The metabolic processes that contribute to the overfat status can begin in utero and continue throughout childhood, placing a significant number of children at high risk of becoming overfat adults. Beyond increasing the risk of chronic diseases, excess body fat can impair immune function and heighten susceptibility to infectious diseases (174). This vulnerability became particularly evident during the COVID-19 pandemic, where overfat individuals were disproportionately affected (175). Excess body fat also contributes to physical impairments, ranging from minor injuries to significant disabilities (176). Moreover, it places an enormous burden on healthcare systems and economies, with global annual costs of overweight and obesity alone projected to reach $18 trillion by 2060 (177).

Determining whether an individual is overfat does not necessarily require expensive or inaccessible technology. While advanced imaging techniques such as Dual-energy X-ray absorptiometry (DEXA) scans provide precise body composition data, their cost and limited availability prevent widespread use for regular monitoring. Instead, waist circumference (WC) serves as a practical and reliable clinical and home measurement for assessing overfat in both adults and children (178), with increased WC independent of concurrent gains in BMI that more likely represents abdominal and visceral fat accumulation (121). A particularly useful metric is the waist-to-height ratio (WHtR), which offers a quick and effective way to assess excess body fat and related health risks, including brain health (179). The WHtR is a valid and useful anthropometric index to assess adiposity, and its prediction of cardiometabolic risk factors associated with NCD, and easily applicable in clinical practice (168, 180, 181). WHtR is also considered more advantageous compared to BMI since its adjustment for height allows a single threshold to be defined which is applicable to the overall population regardless of sex, age, or ethnic group (182) and may be the best anthropometric index when used alone (183). The guideline is simple: waist circumference measured at the umbilicus should be less than half of one’s height.

While increased body fat can occur while consuming any macronutrient beyond caloric requirements, dietary fat has historically been implicated as a primary contributor excess body weight. However, foods that combine both processed fats and carbohydrates are particularly associated with increased body fat and addictive behaviors, more so than unrefined, natural foods (161, 184–186). Studies suggest that weight loss on carbohydrate-restricted diets may be superior to calorie- and fat-restricted diets for adults (187) and adolescents (188). Additionally, very-low-carbohydrate diets high in fat have been shown to effectively reduce excess body fat (189) and are useful for metabolic advantages when compared to low-fat diets (190).

Refined carbohydrates and added sugars alone, even in the absence of dietary fat, are significant contributors to body fat accumulation due to their metabolic effects, including increased insulin secretion and the development of insulin resistance (191). Excessive consumption of sugar-sweetened beverages and other refined carbohydrates has been linked to metabolic syndrome, type 2 diabetes, and other adverse health outcomes, regardless of calorie content (102, 192). Notably, high-sugar and other processed carbohydrate diets can promote adverse metabolic and psychosocial consequences even in individuals without significant increases in body weight (193, 194). These findings emphasize the importance of addressing refined carbohydrate intake as a key strategy for reducing excess body fat to improve brain health.

## Fueling the brain

Impaired glucose metabolism is often a consequence of insulin resistance, and a significant contributor to reduced brain energy (195). The brain demands substantial amounts of energy, primarily derived from glucose, its main ATP source. Ketones and lactate also serve as alternative fuels, supporting neurotransmission, protein synthesis, plasticity, maintenance of membrane potentials, and nearly all other brain functions. At an average rate of 6 kcal/d per billion neurons, the human brain’s ~86 billion neurons require about 516 kcal/day, up to 25% of the body’s total energy expenditure (196). To meet these high energy demands, early humans developed cooking techniques, including the use of fire, which enhanced the digestibility and absorption of nutrients from energy-dense, fatty, and protein-rich animal foods (197, 198). This pivotal evolutionary advancement, occurring between *Homo erectus* and *Homo sapiens*, significantly increased the number of neurons, leading to dramatic enhancements in overall brain size and function (196, 199). The resulting human diet also promoted metabolic flexibility, an adaptation allowing early humans to efficiently use glucose and fatty acids for muscle energy, and glucose and ketones for the brain (200). Metabolic efficiency contributed to reduced disease risk and increased longevity (108, 201). When glucose availability is limited, the brain relies on ketone bodies, such as *β*-hydroxybutyrate and acetoacetate, derived from fatty acid oxidation in the liver, that can cross the blood–brain barrier. These ketones provide a higher ATP yield compared to glucose (84, 85, 202).

Deviating from this ancestral higher-fat, moderate-protein, low-carbohydrate dietary pattern may contribute to today’s risk for nutrient deficiencies, chronic diseases, and reduced brain health (203). While the brain’s metabolic and nutritional needs have remained relatively consistent over millennia, the recent global nutrition transition has significantly altered the human diet, resulting in consumption of significant amounts of sugar and other refined carbohydrates (110). Unhealthy foods—industrial formulations primarily composed of chemically modified substances extracted from whole foods—are designed for long shelf life, convenience, and low perceived cost. They are predominantly composed of highly refined carbohydrates, which can elevate insulin levels, reduce metabolic flexibility, and promote excess fat storage (81, 82, 204, 205). They can also lower brain glucose utilization while impairing the use of ketone bodies as an alternative energy source, promoting brain glucose hypometabolism (75, 84, 85). This metabolic impairment can occur years or decades before the onset of clinical symptoms, including cognitive decline and memory impairment. Individuals with risk factors for Alzheimer’s disease, such as family history, genetic mutations (*presenilin-1*), and the APOE4 gene, are particularly vulnerable (14). Brain glucose hypometabolism can also lead to synaptic dysfunction, neuronal death, and structural thinning in critical brain regions, further impairing brain health (58, 84, 85).

It should be noted that while insulin is produced peripherally in the pancreas, it crosses the blood–brain barrier to regulate glucose metabolism in the brain. The overlapping but distinct actions of peripheral and central insulin highlight their critical but complex roles in metabolic and cognitive health (84, 85, 206).

Numerous brain disorders have shown significant benefits from very-low-carbohydrate diets that increase ketone production, including pediatric epilepsy (297), traumatic brain injuries (207), and chronic neurodegenerative conditions (298) and related oxidative stress (208). These diets are also effective for reducing excess body fat (209).

Low- or very-low-carbohydrate and ketogenic diets are generally higher in fat, with moderate protein and low carbohydrates, with daily carbohydrates comprising only 5–10% of total energy intake or less than 50 g/day (210, 211). Dietary carbohydrate definitions include very-low-carbohydrate or ketogenic diets (≤10% and 20–50 g/day), low-carbohydrate diets (10–26% and 50–130 g/day), and moderate-carbohydrate diets (26–45% and 130–230 g/day) (212). Optimal levels of carbohydrate intake can vary with individual health, e.g., lower levels in those who are overfat and insulin resistant. Despite its clinical success in managing numerous conditions—including Type 1 and Type 2 diabetes, various cancers, autoimmune diseases, and other NCDs—this dietary approach remains underutilized and is not widely endorsed by the medical establishment. This hesitation persists despite the approach’s minimal risks and potential for profound therapeutic benefits (213).

## Expanding brain health

The concept of brain health encompasses all aspects of overall function, independent of underlying pathophysiological processes (2). Brain health reflects enhanced cognitive, behavioral, emotional, and related performance across the lifespan, fostering healthier individuals and societies through improved communication, understanding, decision-making, and overall wellbeing. This includes creativity, which plays a vital role not only in the arts but also in science and virtually all brain activities, driving the generation of novel and practical ideas by organizing established principles to contribute meaningfully to human achievements, both small and large (6, 214). Importantly, creativity supports the mental representation of healthier, future-oriented possibilities rather than remaining anchored in the past (215). Beyond cognitive and emotional performance, brain health relies on critical biological processes, including circulation, ATP generation, cellular repair, and adaptation, among others, to sustain its function and resilience.

Healthy brain performance is often conceptualized through neurocognitive domains, which include:

1. Complex attention: encompasses sustained, divided, and selective attention, as well as processing speed.
2. Executive function: involves goal-oriented and problem-solving behaviors, such as planning, decision-making, working memory, and non-verbal intelligence.
3. Social cognition: pertains to social and emotional behaviors and how individuals relate to themselves and others.
4. Learning and memory: includes both short- and long-term memory functions.
5. Perceptual-motor function: involves visual and motor coordination, critical for physical movements.
6. Language: refers to the production and comprehension of speech and communication (1, 216).

While these categories aid in clinical discussions, they are not mutually exclusive. Impairments in one domain can lead to a broad range of symptoms, often beginning preclinically. Even subtle dysfunctions can diminish performance and pose long-term risks, including increased morbidity, mortality, and rising healthcare costs (12, 84, 85). As such, we advocate for a holistic and transdiagnostic approach to brain health (217), which focuses on addressing shared underlying causes of dysfunction rather than treating isolated conditions, thereby supporting broader and more effective care.

As humans reach full physical and mental maturity, they develop greater capacity to manage the physical, biochemical, and mental–emotional stressors of life (218, 219). Both genetic and environmental factors interact to shape brain health, with ~75% of the variance in cognitive ability throughout life attributed to environmental influences (220, 221).

Lifestyle, shaped by personal choices, external environmental factors, and others, plays a critical role in brain health. For example, access to healthy or unhealthy food options and exposure to food advertising significantly influence dietary habits and associated health outcomes (101, 222, 223). Other factors include physical activity, tobacco use, and alcohol consumption. Moreover, humans instinctively form social groups to foster cooperative behavior, which aids in adapting to stress, enhancing brain function, and improving overall health and fitness (224). This extends to moral behavior, which is intrinsically linked to social cognition and brain health (29). A healthy brain not only enhances its inherent and acquired capabilities but also contributes to the wellbeing of other individuals and society as a whole.

Genetic, lifestyle, and environmental factors contribute to varying levels of resilience to aging, with some individuals maintaining cognitive health longer than others (225). Those with exceptional cognitive abilities despite aging—known as super-agers—exhibit brain health comparable to younger adults. Unlike many of their peers, these individuals can display normal glucose metabolism in regions such as the anterior cingulate cortex and anterior temporal lobes, associated with increased cognitive resilience (226). While the precise mechanisms underlying super-aging and its predictability remain unclear, it underscores the human potential to prevent cognitive decline and sustain brain health well into advanced age.

## Brain injury

Reduced brain health can stem from the progressive loss of neuronal structure and/or function caused by acquired brain injuries. This broad category encompasses any non-congenital, non-hereditary brain disorder (227–229).

Acquired brain injuries result from a wide range of physical, biochemical, and/or mental–emotional insults. Symptoms vary from minor to debilitating and can be classified into two main forms:

1. Traumatic brain injuries (TBI): these injuries are caused by physical trauma to the head and/or neck. Common sources include falls, whiplash, motor crashes, microtraumas, sports injuries, and other events (228, 229).
2. Non-traumatic brain injuries: these are caused by biochemical or metabolic factors that impair brain function, such as:

- Insulin resistance and glucose dysregulation: conditions like hypo- or hyperglycemia and “Type 3 Diabetes” (a term linked to Alzheimer’s disease) are associated with brain injury (39, 230).
- Chronic inflammation: persistent inflammation contributes to neurodegeneration and aging-related diseases (37, 68).
- Excess body fat: overfat conditions correlate with structural brain changes and cognitive decline (68, 231).

Mental–emotional stress also contributes to brain injury by disrupting the hypothalamic–pituitary–adrenal (HPA) axis, leading to neuroplasticity deficits and exacerbating conditions such as major depressive disorder (219, 232, 233).

Reduced brain volume can impair health and be considered a form of brain injury. Research suggests that human brain size may have begun diminishing a few 1,000 years ago (234). As noted, reductions in both gray and white matter volumes have been observed in individuals with excess body fat linked to neuroinflammation, insulin resistance, and reduced glycemic control (66–68, 70, 71), corresponding with the global rise in excess body fat in both adults and children (42, 45). The accumulation of excess body fat and its contribution to early brain atrophy and reduced volume pose significant risks for reduced brain health (68, 72). Moreover, factors contributing to brain injury, such as physical trauma, often coexist with neuroinflammation, which can exacerbate damage and hinder recovery (228). These interconnected mechanisms emphasize the importance of addressing excess body fat and related conditions as part of strategies to maintain brain health and prevent cognitive decline.

### Human performance deficiency

Measurable impairment of brain function can also lead to human performance deficiency, commonly referred to as human error. These deficiencies may involve momentary lapses in attention and effort, potentially resulting in minor mistakes or severe consequences (235). The prevalence of human error is strikingly high across various domains. It accounts for over 60% of accidents in the home, 70–80% of aviation mishaps, and up to 85% of errors in aerospace operations (236). In medicine, human error contributes to more than 50% of complications occurring during major surgeries in the US, leading to an estimated 400,000 potentially preventable adverse events (237).

A recent study found that occupational accidents in secondary industry workers peaked during periods of low blood glucose and accumulated fatigue (238). This aligns with research suggesting that even in healthy individuals, moderate hypoglycemia can significantly impair cognitive functions such as visual and auditory selective attention, attentional flexibility, and information processing speed—while nonverbal intelligence remains unaffected (239).

### Physical activity and the brain

In addition to a healthy diet, physical activity plays a crucial role in reducing major risk factors associated with poor brain health, including hypertension, insulin resistance, and excess body fat, by improving overall fitness (240). However, despite relatively stable physical activity levels, many populations have experienced dramatic increases in excess body fat. For example, among U. S. adults, participation in aerobic or muscle-strengthening exercise increased from 44 to 52% between 1998 and 2014, yet the prevalence of excess body fat increased from 75% to over 90% during the same period (231). This paradox—rising levels of physical activity and increased body fat—is also observed in populations with high levels of exercise, including competitive athletes (241) and military personnel (242).

While exercise is well known to improve cardiovascular fitness, a very-low-carbohydrate, high-fat diet (VLCHF) has been shown to provide even greater cardiovascular benefits in college students compared to high-intensity exercise alone (243). Additional benefits of a VLCHF diet have also been observed in competitive endurance athletes (244). Beyond its physiological benefits, physical activity can enhance brain health, with outdoor exercise providing even greater cognitive benefits than indoor activity (245).

## Discussion

Many national and international health organizations recognize reduced brain health as a critical issue, emphasizing the need for early intervention before the onset of disease and escalating healthcare costs. Despite these concerns, the problem remains unresolved. The overfat pandemic, affecting an estimated 80% of the global population (43, 44), and promoted by the consumption of refined carbohydrates, drives cardiometabolic dysfunction, including insulin resistance, impaired glucose regulation, and neuroinflammation (246, 247), along with various downstream conditions including depression (248). The result is reduced brain health beginning long before the onset of neurodegeneration (46, 47). As increased body fat and reduced brain health are preventable through modifiable lifestyle factors, it has been proposed that the health of those most socially advantaged in society indicates a high level of health that should be attainable for all others, yet a large majority of the global population, and across racial or ethnic groups, is less healthy (117). From a public health, and ethical and human rights perspective, this is unacceptable.

**Figure 3:**
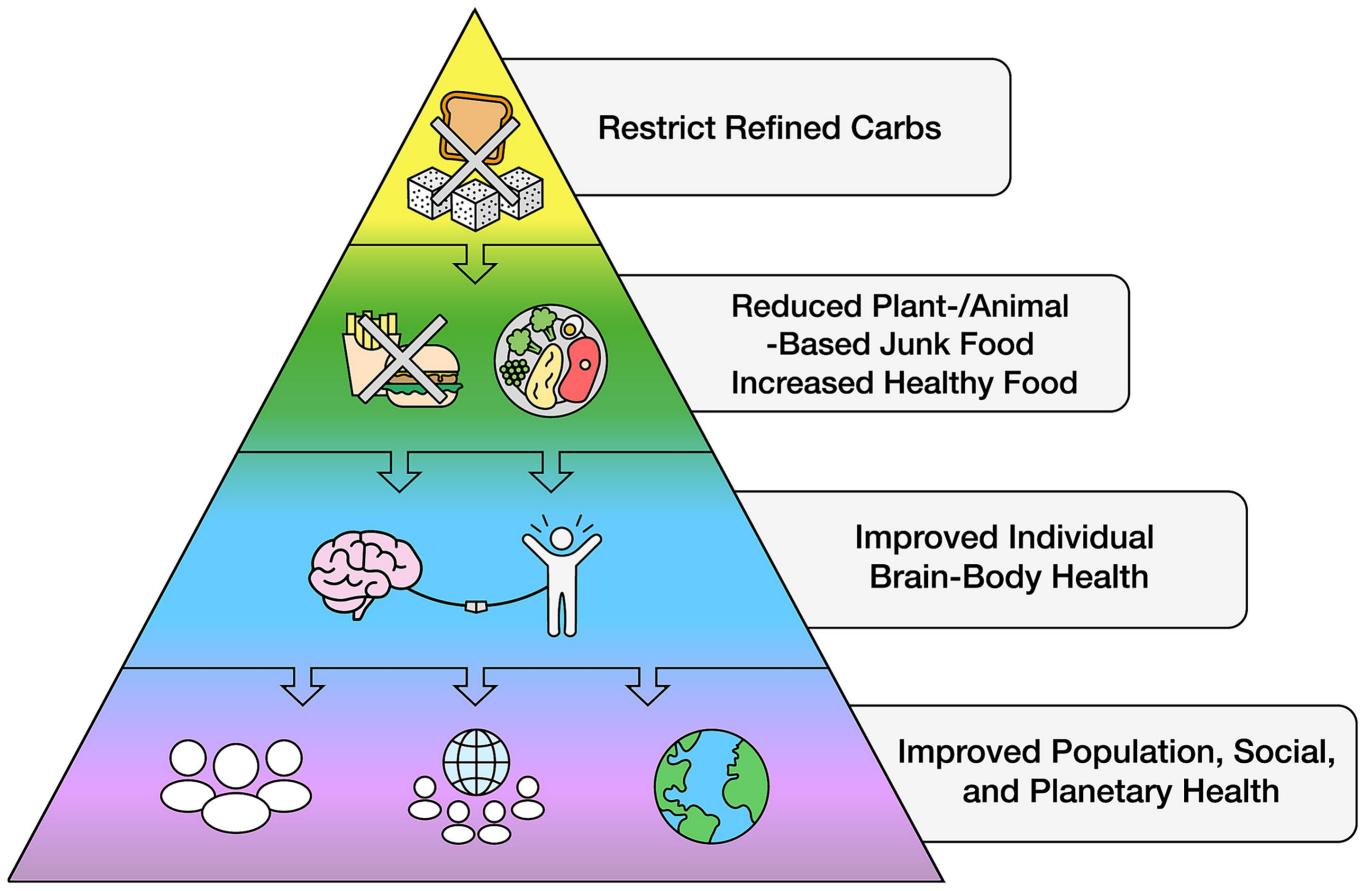
The potential for global health improvements by restricting refined carbohydrates.

The rapid expansion of economic globalization has facilitated the widespread availability of unhealthy food across nearly all nations and socioeconomic barriers (119, 133, 135), displacing healthy foods. Reversing this trend can be cost effective: In addressing the global reduction of NCD alone by one-third through lifestyle modification, it was estimated that an additional US$18 billion annually between 2023 and 2030 could avert 39 million lives and generate an average net economic benefit of US$2.7 trillion—a 19:1 return on investment (249). While some governments and industries argue that limiting access to unhealthy foods could harm national economies, as these refined carbohydrate products contribute significantly to gross domestic product (250), this same argument was previously made against tobacco regulation. However, evidence suggests that reducing unhealthy food sales can be balanced by increased demand for healthier alternatives, which can be distributed and sold through the same retail and supply chains. A global initiative to regulate refined carbohydrate intake could be a crucial step toward reversing the problem of excess body fat and its downstream conditions, improving cognitive function across populations with the potential for rapid, global health improvements (see Figure 3).

A single, targeted lifestyle modification—restricting refined carbohydrates—has the potential for significant impact. For example, just reducing SSB intake has been shown to improve overall dietary quality without replacing other refined carbohydrates and can encourage consumers to make additional spontaneous and healthy changes (251). When replacing all SSB with drinking water it reduced total energy by 200 kcal/d over 12 months (252). Banning SSB sales in the workplace have also been associated with reduced intake and significant reductions in waist circumference within 10 months, with additional improvements by using a short motivational intervention to target employees at higher risk of cardiometabolic health (253).

However, while SSB consumption is a major component of refined carbohydrate intake, it is only part of an overall poor dietary pattern. To maximize recommendations and improve global dietary quality, a comprehensive strategy can more likely better address the current public health challenges (254). Therefore, when developing dietary guidance, other added sugar sources in the diet must be considered while emphasizing reductions of all refined carbohydrates (255). Lessons from alcohol and tobacco regulation provide valuable insight in reducing refined carbohydrates, as public health policies that restrict access and availability—such as taxation—have repeatedly shown effectiveness in reducing harmful substance use (140, 246, 256, 257).

### A science-based ancestral dietary pattern

A viable strategy to effectively address the problem of reduced brain health requires a clear scientific approach independent of political and commercial influences. One consideration is to base healthy dietary habits on those that sustained human evolution for millions of years—a natural food-based eating pattern that is higher in healthy fats, moderate in protein, and low in carbohydrates without refined carbohydrates (25, 199, 201, 244, 299–308). Nowadays, similar approaches with well-known benefits are found in the ketogenic diet, the Mediterranean and Mediterranean-DASH Intervention for Neurodegenerative Delay (MIND) diet, and other low- and very-low-carbohydrate and anti-inflammatory approaches, with extensive evidence that these diets enhance brain health and reduce excess body fat (47, 70, 258–262). The potential benefits of significantly reducing refined carbohydrates have not yet been effectively translated into organized global dietary guidelines. Like other government guidelines there may be several reasons for this gap. For many consumers, the sheer volume of dietary information—often contradictory and coming from multiple sources—creates confusion and frustration, negatively impacting attitudes, beliefs, and behaviors (261, 262). While public trust in science is fundamental to improving population health, distrust of scientific information remains a major barrier (263). Yet, when the addictive properties of unhealthy food are framed similarly to those of tobacco, awareness, belief, and education about these risks can lead to increased public support for regulation (186, 264, 265).

### The global push to regulate refined carbohydrates

Similar public health measures restricting refined carbohydrates are already being implemented on relatively small scales in various regions with measurable success. These include taxes on SSB in countries like Mexico and the U. S. leading to a sustained reduction in consumption (266), with workplace bans on SSB significantly improving employee health outcomes (253), and others discussed above. Included are policies that restrict or ban unhealthy foods in retail stores, restaurants, schools, and vending machines, with consumer compliance increasing when product prices rise. These and other approaches generally mirror successful restrictions placed on tobacco, demonstrating that reducing availability directly reduces consumption and, ultimately, improves health outcomes (246, 256, 257, 267).

While this approach could reduce refined carbohydrate consumption for all consumers, the effects can differ by socioeconomic status. While increased tax revenue may come from high income consumers, when viewed as a percentage of total household expenditures, lower income consumers may assume more of the financial burden (268). However, larger health benefits often accrue to low-income consumers because of their stronger response to price changes, the potentially larger financial burden could also be mitigated by a pro-poor use of the generated tax revenues (directing more resources toward them). While carefully designed and implemented approaches can be successful and fair to all socioeconomic groups, this complex economic issue goes beyond the scope of this paper.

**Figure 4:**
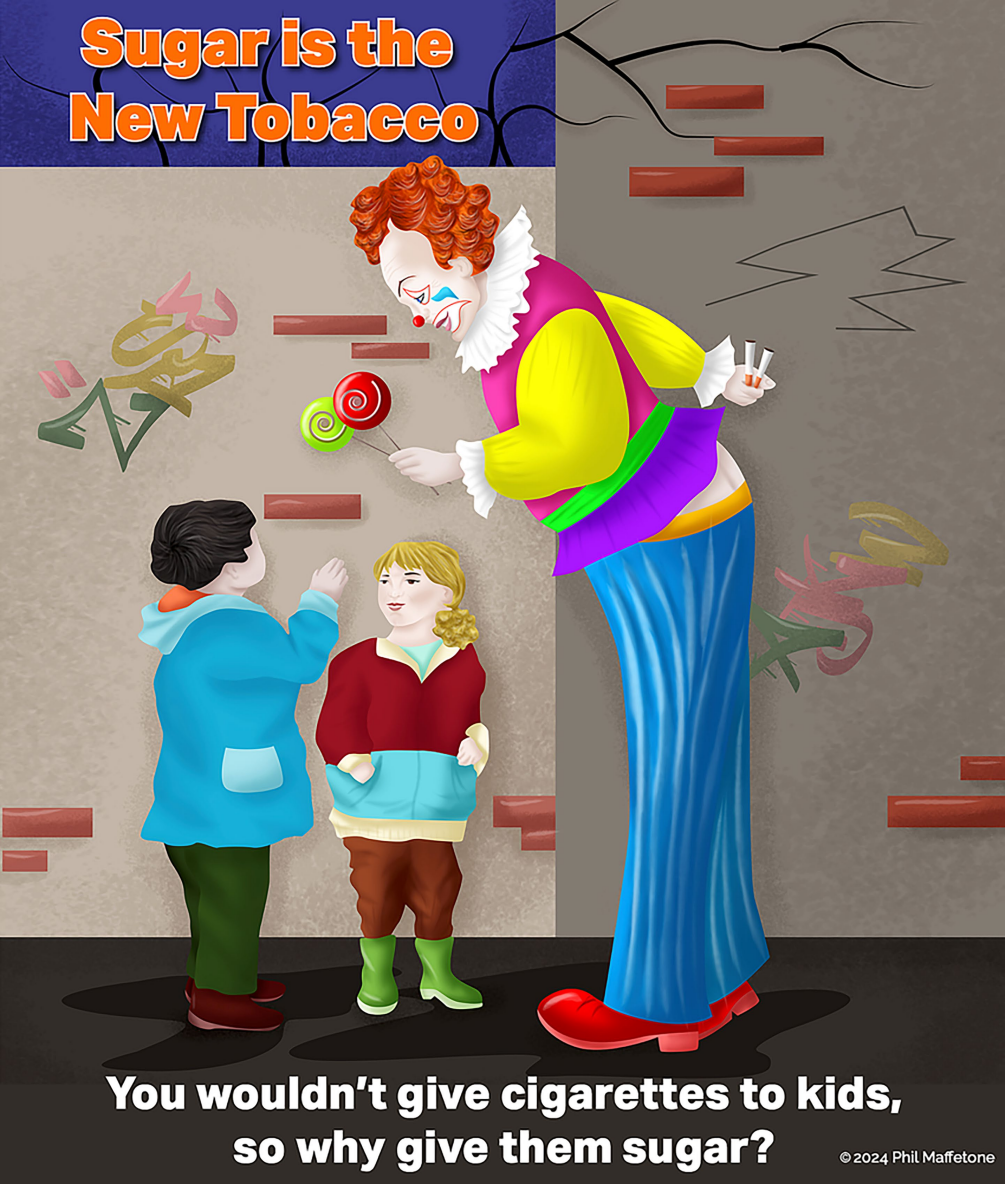
Sugar is the new tobacco. Reproduced from “Sugar is the New Tobacco’’ by Phil Maffetone (https://maffetonemusic.com).

Understanding past public health errors—where many health organizations and governments only passively discouraged tobacco use, allowing its prevalence to persist for decades—the modern phrase “sugar is the new tobacco” serves as a fitting call to action in reducing the consumption of unhealthy foods (269–272). While this comparison of two different but harmful substances should not weaken the rationale for adopting related regulatory framework, some of the successful policies and regulations that curtail tobacco use could similarly be applied to refined carbohydrates, including methods that limit public access and restrict marketing and advertising (102, 267, 273, 274) (see Figure 4).

Importantly, consumer decision-making is associated with food choices (275) and must be considered along with the biological mechanisms affecting brain health discussed above, including those associated with addiction and eating behavior (160). While these are all associated with brain health, powerful influences come from companies advertising and marketing products, and governmental recommendations which are strongly affected by politics and lobbying. A primary factor that can significantly influence food purchases and consumption is cost.

Given these considerations, implementing not one but a combination of factors associated with taxation, lobbying, marketing restrictions, financial incentives, and other regulatory measures can help reduce the consumption of unhealthy foods. As in past public health challenges—such as tobacco control, drunk driving prevention, and HIV intervention—effective initiatives can successfully overcome key barriers to change, including socioeconomic status, education, income, and environment (3).

Various strategies to help improve brain health are summarized below.

### Taxation

One of the most important strategies to reduce the consumption of refined carbohydrates is increasing their cost, often through taxation along the supply chain (276, 277). As evidence suggests that the resulting higher costs of unhealthy food and beverages lead to a measurable decline in consumption, this approach could include raising taxes on unhealthy foods while lowering taxes on healthier options to create incentives for better dietary choices. For this to be effective, the price adjustments must be substantial enough to influence purchasing behavior (278). This could also address the physical food environment issue (related to food insecurity discussed above): many people may not have access to healthy foods. Easier access to healthy and affordable food occurs by replacing the available unhealthy foods with healthy options in the same retail and wholesale food distribution areas.

### Food labeling

Food ingredient labeling and product information, including those in advertising and marketing, use a variety of terms and definitions that differ across government and regulatory agencies worldwide. Many products labeled as “whole grain” contain high percentages of refined carbohydrates, making labeling inconsistent and potentially misleading for consumers (96, 97). Such discrepancies in labeling can obscure product quality and nutritional value. Improving food labeling and advertising regulations—particularly through the adoption of standardized definitions—may help enhance consumer understanding to limit inadvertent consumption of refined carbohydrates.

### Food subsidies

In addition to taxes, significant health gains and cost savings can be made by addressing food subsidies (279, 280). The global junk food industry is supported by governments that subsidize crops used to produce unhealthy food ingredients. Without these subsidies, unhealthy food costs would rise. These subsidies can instead be transitioned to support healthier food production.

### Lobbying

Food industry lobbying efforts have proven more successful at shaping consumer behavior than public health campaigns. Much like the tobacco industry, the sugar industry and its political allies have strongly influenced policies and public perception (102, 281). Since the 1950s, the sugar industry lobby—a powerful Washington, DC-based trade association—has actively misrepresented its products, shifting blame onto dietary fat to divert attention from the harmful effects of sugar (282). Another major lobbying success occurred in 1966, when the National Institute of Dental Research aimed to eradicate dental caries. Rather than acknowledging sugar’s role, the industry redirected focus away from sugar consumption as the primary cause of cavities (283). This was despite the strong scientific consensus that sugar contributes to tooth decay and overall poor oral health through systemic effects and direct reduction of oral pH (284). Today, the food industry continues to shape dietary choices through misinformation, including on social media, where misleading content further encourages unhealthy eating habits (285).

### Advertising and marketing restrictions

Policies that limit public advertising and marketing of unhealthy foods are essential for improving public health (273). Beyond traditional ads on TV, radio, billboards, and the Internet, unhealthy food marketing infiltrates stores, shopping centers, public transportation, and even community spaces. A particularly concerning strategy is athletic sponsorship, where junk food companies serve as major sponsors of public events worldwide, targeting both adults and children. This is evident in local, amateur, and professional sports, including the Olympic Games, where these brands associate their products with athletic excellence despite their negative health effects.

### Litigation

The legal system has long played a crucial role in promoting and protecting public health. Similar to the successful lawsuits against the tobacco industry, litigation against unhealthy food companies could be a viable strategy for change (267). Tobacco lawsuits, particularly those led by U. S. attorneys general, significantly shifted public attitudes about smoking. A similar legal approach targeting junk food manufacturers could raise awareness, hold corporations accountable, and drive policy change.

### User financial incentives

While taxation can discourage unhealthy food consumption, it may be less effective than strategies that actively engage individuals in improving their health (250). One proven approach is offering financial incentives for making healthier choices. Studies show that paying individuals to adopt better health habits can lead to significant benefits (286–289). Despite the upfront costs, a carefully monitored program can lead to long-term savings by reducing healthcare expenditures on treatments and medications. Similarly, corporate wellness programs yield up to six times the company’s investment (290). Schools implementing free, high-quality meal programs have shown reduced obesity rates among children (291, 292), highlighting the potential of financial incentives in promoting healthier choices at both individual and systemic levels.

To support these and other initiatives, public education, which may not be effective on its own, should be delivered through simple, straightforward, consistent, and ongoing campaigns. These efforts should include clear product descriptions, school-based education, and scientific-based guidelines from unbiased health organizations (293, 294). Additionally, tailored strategies should be developed to align with the socioeconomic conditions of specific regions, ensuring that interventions are both effective and culturally relevant. A comprehensive global brain health initiative must focus on reducing the availability and consumption of unhealthy foods while simultaneously fostering improved dietary habits and overall brain health.

## Conclusion

Optimal brain health is essential for the wellbeing of individuals, societies, and the environment. The global overfat pandemic signals widespread and worsening cognitive and cardiometabolic health issues largely driven by consumption of sugar and other refined carbohydrates. Drawing from successful public health strategies used to combat tobacco, regulatory measures such as taxation, marketing restrictions, litigation, and financial incentives offer a viable path to reducing refined carbohydrate consumption. Implementing these strategies alongside targeted education and policy reform can significantly help lower excess body fat, enhance brain function, curb chronic disease, and alleviate the growing burden on healthcare systems and economies worldwide. A proactive, science-driven approach is crucial to reversing these trends, fostering a healthier global population and long-term societal wellbeing.

## Data Availability

The original contributions presented in the study are included in the article/supplementary material, further inquiries can be directed to the corresponding author.
